# Nursing Practice in Rondônia (1946-1976): a historical study based on the Alto Madeira Newspaper

**DOI:** 10.1590/0034-7167-2025-0309

**Published:** 2026-10-19

**Authors:** Phernando Pereira dos Santos, Jackeline Felix de Souza, Maria Lígia dos Reis Bellaguarda, Deybson Borba de Almeida, Maria Sagrario Gómez Cantarino, Maria Angélica de Almeida Peres

**Affiliations:** I Universidade Federal do Rio de Janeiro. Rio de Janeiro, Rio de Janeiro, Brazil; II Universidade Federal de Rondonópolis. Rondonópolis, Mato Grosso, Brazil; III Universidade Federal de Santa Catarina. Florianópolis, Santa Catarina, Brazil; IV Universidade Estadual de Feira de Santana. Feira de Santana, Bahia, Brazil; V Universidad de Castilla-La Mancha. Toledo, Spain

**Keywords:** Nursing, Nurse Practitioners, Periodical, Nursing Care, History of Nursing., Enfermería, Enfermeras Practicantes, Publicación Periódica, Atención de Enfermería, Historia de la Enfermería.

## Abstract

**Objectives:**

to analyze the work of nursing professionals in Rondônia between 1946 and 1976.

**Methods:**

a socio-historical, qualitative, and documentary study based on the analysis of articles published in the Alto Madeira newspaper. The documentary research followed three stages: pre-analysis, organization, and data analysis, with interpretation grounded in the socio-historical context of nursing in the state.

**Results:**

a progressive increase in professional engagement in nursing was observed, with emphasis on hiring processes, work settings, advertisements, and events. Female visibility was significant, with qualities such as experience and competence being highlighted. Professional activities included caregiving, medical support, teamwork, and health education.

**Conclusion:**

despite precarious working conditions and the absence of nursing training programs during the period, the work of these professionals was documented by the Alto Madeira newspaper, which served as an important historical source on the profession and the consolidation of nursing in Rondônia, especially after the establishment of the Regional Nursing Council in the Amazon region.

## RESUMEN

**Objetivos:**

analizar la actuación de los trabajadores de Enfermería en Rondônia entre 1946 y 1976.

**Métodos:**

estudio sociohistórico, cualitativo y documental, basado en el análisis de artículos del periódico Alto Madeira. La investigación documental siguió tres etapas: preanálisis, organización y análisis de los datos, con interpretación anclada en el contexto sociohistórico de la enfermería en el estado.

**Resultados:**

se observó un aumento progresivo en el compromiso profesional de la enfermería, con énfasis en contrataciones, lugares de actuación, anuncios y eventos. La visibilidad femenina fue significativa, destacándose cualidades como experiencia y competencia. La actuación abarcó cuidados, apoyo médico, trabajo en equipo y educación en salud.

**Conclusiones:**

a pesar de las condiciones precarias de trabajo y de la ausencia de cursos de enfermería en el período, la actuación de estos profesionales fue registrada por el periódico Alto Madeira, que se constituyó en una importante fuente histórica sobre la profesión y la consolidación de la enfermería en Rondônia, especialmente después de la creación del Consejo Regional de Enfermería en la región amazónica.

## INTRODUCTION

The production of studies on the History of Nursing in the Northern region of Brazil has been growing slowly, a fact attributed to the characteristics of the region’s own health development as well as its population profile^(1)^. Indigenous culture, which for a long time was predominant in the North, maintained its health practices within the villages, avoiding the use of services in urban centers, while the urban population grew more significantly throughout the 20th century.

The territory of Rondônia previously belonged to the states of Amazonas and Mato Grosso and, in 1943, was designated as the Federal Territory of Guaporé, later becoming the Federal Territory of Rondônia in 1956. It became a state in 1981. This process had significant impacts on population growth and economic activities, resulting in environmental challenges such as deforestation and pressure on natural resources^(2)^. Likewise, it required the organization of public health services and the presence of nursing personnel.

The development of health care in Rondônia was influenced by several factors. Between the 1900s and 1950s, the challenging geographic and climatic conditions of the Amazon region, combined with limited access to the area, likely affected the availability of health services^(3)^. Indigenous and riverside populations faced specific challenges when economic exploitation through latex extraction began^(4)^.

At the beginning of the 20th century, during the Rubber Boom, the construction of the Madeira-Mamoré Railway (“*EFMM*”) led to the establishment of the first hospital in the state, the Hospital da Candelária, in 1907. Initially created to serve *EFMM* workers and the foreign professionals responsible for the project, it operated until 1930, when the railway was deactivated^(5)^. With its closure, the São José Hospital, inaugurated in 1929, became the main reference for healthcare services, marking more than five decades of significant contributions to health care in the region^(5)^.

A previous study examining nursing in Rondônia from 1907 to 1930 showed that the profile of Nursing workers in the state consisted of civilians, many of them immigrants from countries such as Morocco, Lebanon, Syria, Japan, China, India, Barbados, Bolivia, Italy, and Germany, as well as Brazilians from various regions of the country, mostly from the Northeast. They worked in the fight against infectious diseases that afflicted the region, following the guidance of physicians^(5)^.

Among the developments linked to Rondônia’s economic growth after the 1940s were the construction of highways such as *BR-364*, the emergence of new healthcare facilities, and the creation of the *Conselho Regional de Enfermagem* (“*Coren*”, Regional Nursing Council) in the 1970s, which established legal requirements, including ethical standards, training, and competencies for Nursing practice^(6)^.

The knowledge produced, in addition to disseminating the history of nursing in a state where such scholarship is scarce, will contribute to Nursing education by becoming teaching content capable of bringing visibility to nursing in the North of Brazil. The work carried out by these professionals contributes to raising the region’s human development index through the provision of care and the promotion of health.

## OBJECTIVES

To analyze records of the presence and activities of nursing personnel during the urban development of the state of Rondônia.

## METHODS

### Ethical aspects

This study followed the Standards for Reporting Qualitative Research (SRQR). As it involved sources made publicly available by the National Library, submission to and approval by a Research Ethics Committee was not required.

### Type of study

Descriptive, qualitative, exploratory research, grounded in Social History.

### Context and setting

The spatial scope encompasses the former Federal Territory of Guaporé, located in the Northern region of Brazil, created in 1943 through Decree No. 5,812 of that same year. In 1956, it became the Federal Territory of Rondônia, which was later elevated to the status of the State of Rondônia by Law No. 41 in 1981.

The study’s time frame, 1946-1976, corresponds to the first and last mentions of nursing workers in the Alto Madeira newspaper during the period in which it circulated as a periodical of the Federal Territory - first of Guaporé (1943-1955) and later of Rondônia (1956-1980). The decision was made to use the current name (Rondônia) throughout the text to facilitate the reader’s identification of the region under study

### Data sources

The documentary corpus consisted of articles from the Alto Madeira newspaper, created in 1917 as a biweekly publication managed by the merchant João Soares Braga, a Portuguese national who also served as the municipality’s treasurer due to his accounting skills. An analysis of its first issue classified it as a newspaper with an open thematic scope^(7)^. Letters sent to the newspaper were selected by its manager, with the notice that “the editorial board would not be responsible for opinions expressed by its contributors”^(7)^. It circulated until 2017, the year of its centenary, when its final edition was published.

### Data collection procedures

The documentary research method was chosen for the treatment of sources and data, consisting of three phases: pre-analysis (selection and compilation of documents), organization of the material (distribution of documents and extraction of meaning and significance from the messages contained in the sources), and analysis of the results (which involves socio-historical contextualization, contemporary connections, and respect for the historical and spatiotemporal context)^(8,9)^.

Searches of all editions were conducted using documents available on the Brazilian Digital Newspaper Library website, based on the descriptors “nurse” (*enfermeira*), “male nurse” (*enfermeiro*), and “nursing” (*enfermagem*). Fifty editions of the Alto Madeira newspaper (RO) published in the 20th century were identified, with 39 sources meeting the research criteria. Inclusion criteria consisted of articles about nursing workers in Rondônia and articles of regional or national interest related to the profession. Exclusion criteria included articles reporting nursing-related events occurring in other states or countries, duplicate articles, mentions of nursing workers outside the work context, and prayers or short stories in which the keywords appeared.

The data were organized using Microsoft Excel^®^, including information such as source characterization (date of collection, date of the recorded information, author of the record, news topic, bibliographic reference) and any other information related to nursing during the period. Based on the spreadsheet, figures were prepared to facilitate the presentation of the findings.

### Data analysis procedures

The analysis encompassed three interrelated dimensions: the means of communication, whose evaluation considered aspects such as editorial stance, credibility, and discursive strategies; the reader’s interpretation, in which meanings emerged anchored in sociocultural repertoires and individual perspectives; and the social context of news production, which clarified the influence of historical, political, and economic factors on the construction and circulation of information^(10)^.

Indirect sources, such as books and scientific articles, were used for interpretation and discussion, taking into account the context of Rondônia and of Nursing during the study period, establishing relationships of agreement, understanding, or disagreement among the facts^(11)^.

## RESULTS

Thirty-nine individuals were mentioned by name in the articles of Jornal Alto Madeira (RO) between 1946 and 1976, thirty-five of whom were Nursing workers and two were prominent figures in the history of the profession (Florence Nightingale and Anna Nery). Of these, 23 were mentioned in the 1940s and 1950s (Figure 1) and 12 in the 1960s and 1970s (Figure 2).

**Figure 1:**
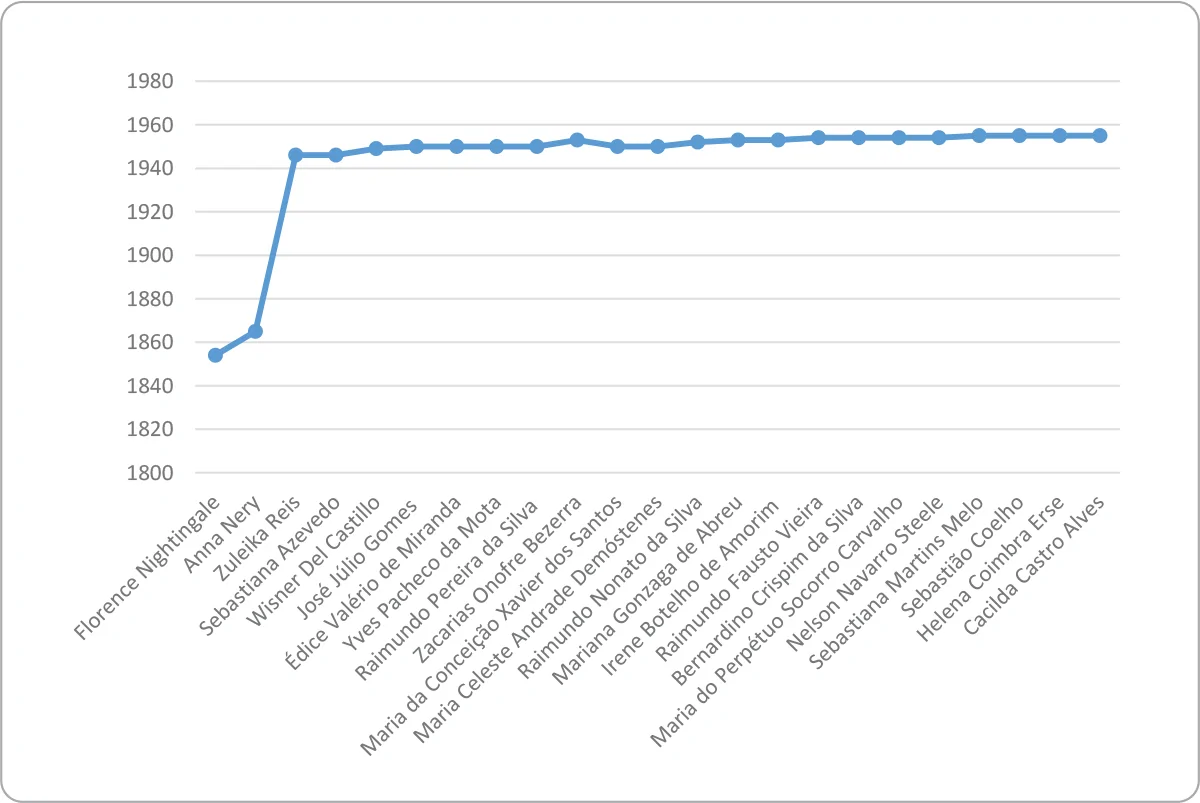
List of Nursing workers mentioned in Alto Madeira newspaper in the 1940s and 1950s, Porto Velho, Rondônia, Brazil, 2025

*Source: Alto Madeira Newspaper. Rondônia (RO): Brazilian National Digital Library (BR); 1948 [quoted Feb 16th, 2025]. Available at: https://bndigital.bn.gov.br/hemeroteca-digital/.*

**Figure 2:**
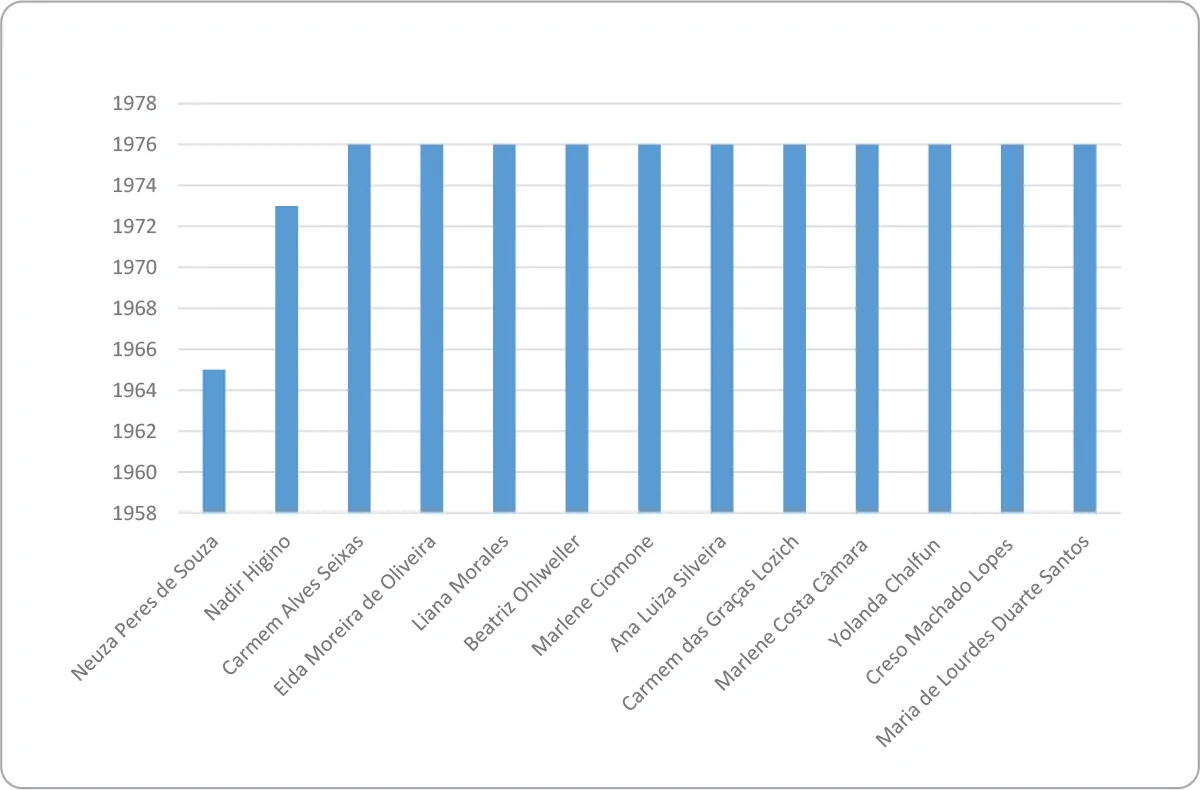
List of Nursing workers mentioned in Alto Madeira newspaper in the 1960s a 1970s, Porto Velho, Rondônia, Brazil, 2025

*Source: Alto Madeira Newspaper. Rondônia (RO): Brazilian National Digital Library (BR); 1960-1970 [quoted Feb 16th, 2025]. Available at: https://bndigital.bn.gov.br/hemeroteca-digital/.*

The year 1976 showed the highest number of articles mentioning nursing in the newspaper texts. Regarding gender, 12 men (34.29%) and 23 women (65.71%) were cited as Nursing personnel. This distinction is important because it highlights the presence of men working in nursing even after the implementation of Modern Nursing in the country, which until then had been an exclusively female domain.

The categories mentioned in the articles included Nurse, Nursing Assistant, Nursing Technician, Practical Nurse, Registered Nurse, Nursing Trainee, Health Assistant, and Nursing Student.

As for the places where nursing practice occurred, the sources made it possible to identify several services. The Health Division of the Territory of Guaporé/Rondônia hired nurses who were assigned to public services; therefore, it was not included in Figure 3, which refers only to the health services mentioned in the articles included in the study.

**Figure 3:**
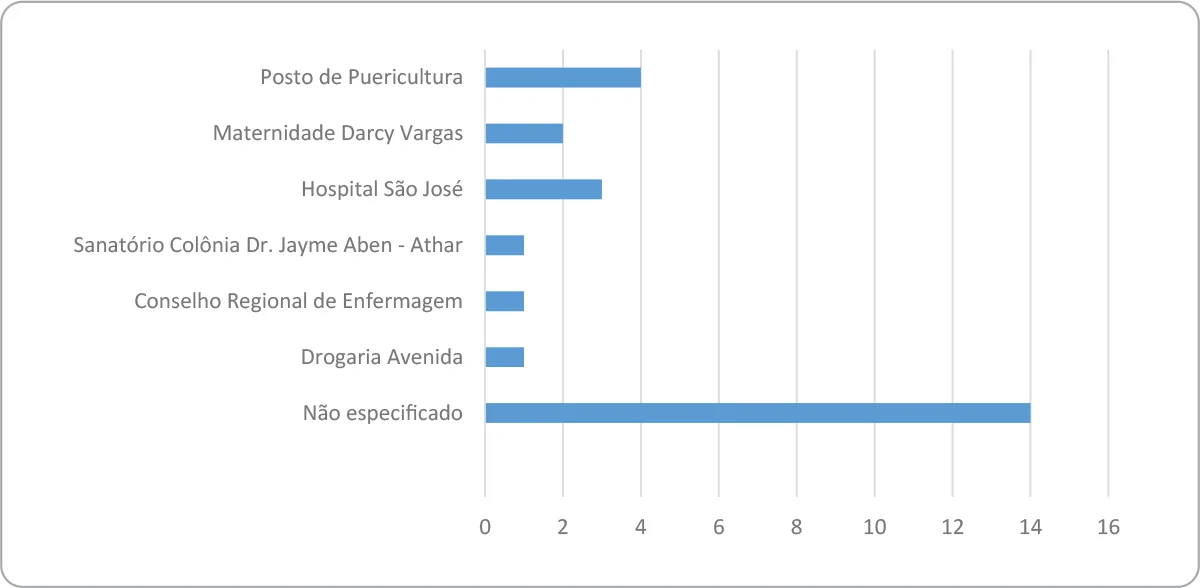
Workplaces of nursing workers in the state of Rondônia mentioned in Alto Madeira newspaper between 1946 and 1976, Porto Velho, Rondônia, Brazil, 2025

**Source:** *Alto Madeira Newspaper. Rondônia (RO): Brazilian National Digital Library (BR); 1946-1976 [Quated Feb 16^th^, 2025]. Available at: https://bndigital.bn.gov.br/hemeroteca-digital/.*

The locations identified as practice settings for nursing workers were the following institutions in Porto Velho: São José Hospital, the Childcare Post, Darcy Vargas Maternity Hospital, Dr. Jayme Aben-Athar Colony Sanatorium, and Avenida Drugstore.

The contexts in which nursing personnel were mentioned varied, including functions performed, appointments to positions and services, birthdays, announcements, commendations, welcomes, among others. The first news item, from 1946, refers to a welcome message for Nurse Zuleika Reis, who had returned from the Federal Capital, where she had taken leave. In the article, she was described as a ‘competent nurse of São José Hospital’^(12)^. In the same year, an advertisement for a nurse was published, in which Sebastiana Azevedo, ‘certified by the Territory’s Health Department’, offered her services at home, on any day, at any time, and free of charge to poor individuals who could not travel to health services^(13)^.

Regarding articles in which references to nursing were not nominal, their content addressed health education, staff composition, regulation of the profession, among other topics. Notably, as presented in Figure 4, one article titled ‘Your Child’ featured the image of a nurse dressed in white and wearing a cap, portrayed while conducting a health education activity.

**Figure 4:**
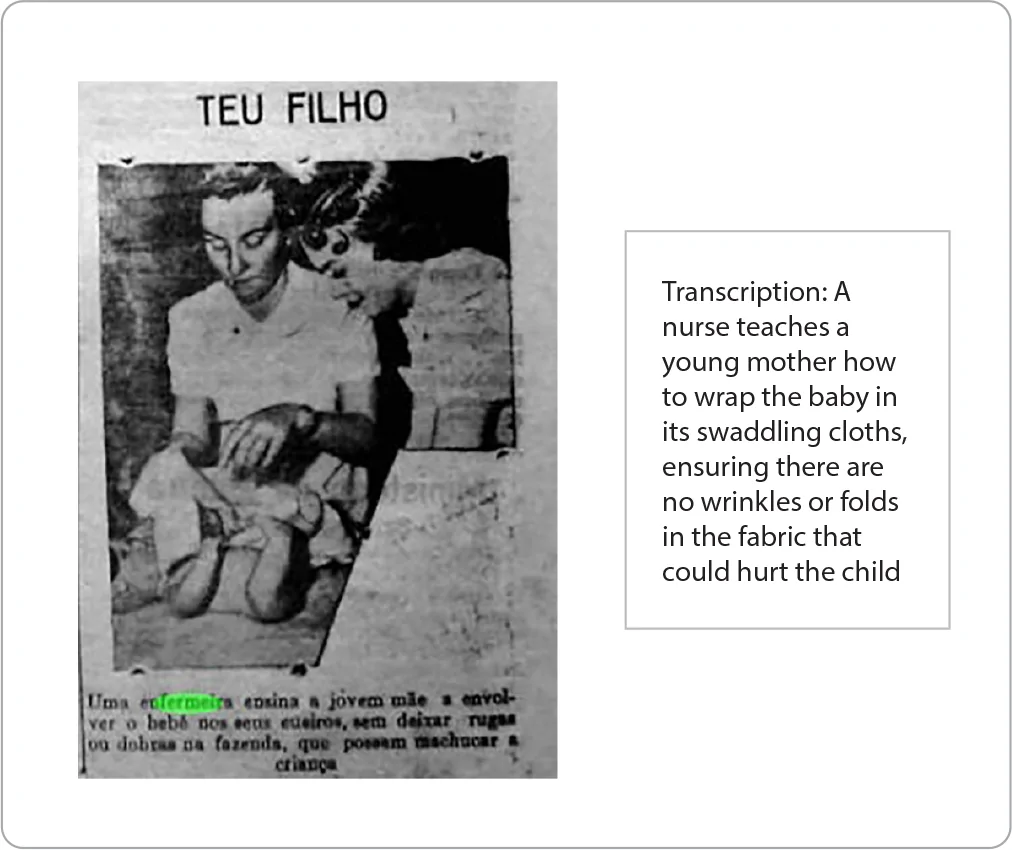
Article ‘Your Child’ published in Alto Madeira Newspaper, Porto Velho, Rondônia, Brazil, 1948

*Source: Alto Madeira Newspaper. Rondônia (RO): Brazilian National Digital Library (BR); 1948 [quoted Feb 16th, 2025]. Available at: https://bndigital.bn.gov.br/hemeroteca-digital/.*

An advertisement from Avenida Drugstore promoted the ‘application of injections at affordable prices by a competent nurse’^(14)^.

Another news item highlighted in Alto Madeira newspaper in 1976 reported the arrival of nursing students from the Federal University of Rio Grande do Sul (UFRGS) to participate in a national operation of the Rondon Project, which aimed to provide reinforcement in the health sector. The students worked in 23 localities from Guajará-Mirim/RO to Vila Bela/MT. The article stated:

‘The young nurses, in addition to administering medications and dressings, provided guidance in each community to individuals capable of taking on such activities, and instructed mothers on childcare issues’^(15)^.

Nursing Week was featured in Alto Madeira newspaper on May 12^th^, 1976, in an article titled ‘Governor presides over the opening of Nursing Week.’ The opening ceremony, led by Governor Humberto da Silva Guedes, took place at the Carmela Dutra Normal School at 8 p.m. The Week was organized by the Territory’s Department of Health and Social Action and by the Nursing Council. The article announced that on May 13^th^ a lecture on the Administrative Organization of the Department of Health would be delivered by Dr. Humberto Vieira Leal, and that on May 15^th^ Nurse Marlene Clomone would present the topic ‘The Participation of Nursing in the Health Plan of Rondônia’^(16)^.

Throughout Nursing Week, articles were published consecutively in the editions of Jornal Alto Madeira. On the 13^th^, the article carried the same title as the previous one, ‘Governor presides over the opening of Nursing Week,’ describing the ceremony and providing details of the program, which included lectures, the composition of the Board of the Regional Nursing Council (*Coren*), a service, a mass, the preparation of studies, and the Rural Integration Program of the Rondônia Health Plan^(17)^.

On the 14^th^, the article was titled: ‘Governor highlights the role of the Nurse.’ On the 15^th^, ‘The Nurse is also important in the patient’s recovery,’ and in the Sunday and Monday edition, May 16^th^ and 17^th^, the article ‘Nursing’ was published, in which the authorities present at the opening ceremony were mentioned by name, along with a summary of their speeches. Notable attendees included the Governor of the Territory, the Mayor, the Secretary of Health and Social Action, the Secretary of Education, the Director of the Human Resources Department of the Superintendency for the Development of the Center-West Region (SUDECO), and the Director of Complano (Planning and Organization Consultancy)^(18)^.

The speeches cited were: that of the Secretary of Health and Social Action, Jacob Atallah, ‘who spoke about the importance of Nursing within the context of medical activity, as an indispensable supplement to this work’; that of Consultant Carmen das Graças Lozich, ‘representative of the Regional Nursing Council, who clarified the regulation of the Nursing profession’; that of Complano’s Nursing Consultant, Marlene Costa Câmara, ‘who highlighted the work of the Nurse, which dignifies the profession, emphasizing the condition of the woman nurse, worthy of recognition’; and finally, that of Governor Humberto da Silva Guedes, who ‘closed the ceremony by emphasizing the work of the nurse as a service grounded in respect for human dignity, where affection and comfort for those in need of care are fundamental’^(19)^.

Following Nursing Week, from May 18^th^ to the 20^th^, 1976, articles invited the public and announced the closing of the event, as illustrated in Figure 5.

**Figure 5:**
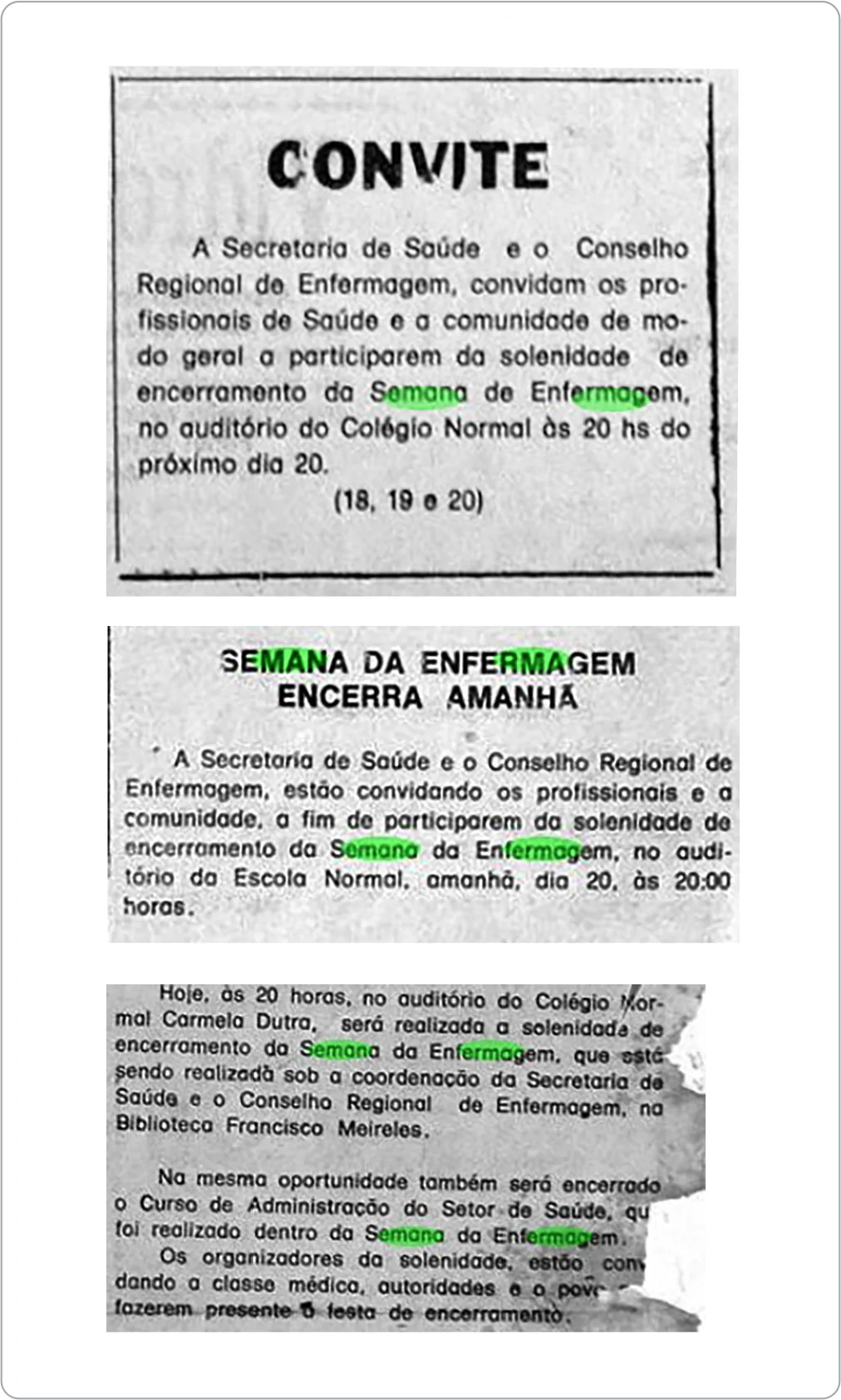
Articles about the closing of Nursing Week in Rondônia, published from May 18^th^ to the 20^th^, 1976 editions of Alto Madeira newspaper, Porto Velho, Rondônia, Brazil, 1976

*Source: Alto Madeira newspaper. Rondônia (RO): Brazilian National Digital Library (BR); 1976 [quoted Feb 16th, 2025]. Available at: https://bndigital.bn.gov.br/hemeroteca-digital/.*

On the days when the invitation to the closing of Nursing Week was published, additional articles about nursing also appeared. One highlighted information about a bill proposed by Congressman Nelson Carneiro, which would add provisions to Law No. 2,604/1955 regulating the practice of professional nursing in Brazil, stating that ‘practical nurses who have been working for more than five years, after a knowledge test, will receive - if approved - the official nursing diploma.’ Another article reported that a Health Administration Course was offered as part of Nursing Week and extended an invitation to the medical community, authorities, and the public to attend the closing session^(20)^.

Subsequently, the article published on May 21 was titled ‘Governor presided yesterday over the Closing of Nursing Week,’ and finally, on May 22, the column ‘Highlighting’ reported that Dr. Leônidas Rachid Jaudy hosted nurses and guests at his ranch to celebrate Nursing Week, with the presence of the Secretary of Health, Dr. Jacob Freitas Atallah.

## DISCUSSION

Throughout the twentieth century in Brazil, the nursing profession underwent a remarkable evolution in terms of professional organization, with the establishment of formal training programs such as the Nursing Training Course in São Paulo in 1912, the Volunteer Nurses Course in Rio de Janeiro in 1914 - both offered by the Brazilian Red Cross (CVB) - and the Nursing Course of the National Department of Public Health in 1923, in the capital, Rio de Janeiro. In the 1930s, the nursing assistant course was introduced, followed in the 1960s by the nursing technician course. Graduate-level nurses (higher education) created an Association and gradually assumed leadership, management, and decision making roles in public health and hospital institutions, while non credentialed religious workers became increasingly distant from care activities. This movement was more pronounced in the Southeast region and culminated in the regulation of the profession in the 1970s and 1980s^(21,22)^.

It is important to highlight that Alto Madeira newspaper published articles in which individuals were identified as nurses according to their employment status rather than their level of training. Between 1931 and 1949, Decree Law No. 20,109/1931, which regulated the professional practice of nursing in Brazil, was in force. Article 1 of this decree stated the following:

*Art. 1º Only those may use the title of registered nurse or the corresponding initials: a) professionals who hold a diploma from official nursing schools or from schools considered equivalent under this law; b) professionals who, having graduated from foreign schools recognized by the laws of their country, qualify before the competent examining board or are hired by the federal or state administration* ^(23)^.

In fact, this occurred later in the Northern region, since, in the absence of a nursing training program until 1944 - when the Pará School of Nursing was created - the term ‘nurse’ continued to be used for any nursing practitioner or hospital aide, whether they had completed a preparatory course or not, especially in places where no nursing school existed.

Regarding gender, there is a predominance of women when the newspaper refers nominally to nurses. Two female workers were mentioned in 1946, increasing to ten in 1976. Male presence appears only incipiently, with one worker cited in 1955 and one in 1976, reflecting a significant gender disparity. In 1955, five male workers were mentioned. This scenario highlights the historical association between nursing and the social role of women. The increase in women in the profession reflects both the growing demand for health professionals and the broader entry of women into the labor market, driven by social and economic changes of the period, and even more so by the implementation of Modern Nursing in the country, whose initial characteristic was female exclusivity.

In 1946, an advertisement offering the services of a ‘registered nurse’ in Rondônia was published. However, this term was used as an identity marker for nurses trained in schools that followed the standardized national curriculum established in 1931, which requires clarification, since the historiography of nursing does not record the existence of a standard nursing school in the Northern region at that time. Nevertheless, it is known that the newspaper aligned itself with the hegemonic thinking of the period in which holding a diploma was not the main requirement for becoming a nursing worker in places where such personnel were scarce.

Regarding technical and auxiliary professionals, the results indicated a greater number of references to nursing assistants in 1950. Although the nursing assistant course in Brazil was officially regulated in 1949, the position of assistant already existed in health services^(24)^.

In the late 1960s, colonization measures were implemented, leading to the gradual occupation of the Northern region, during which Rondônia experienced significant economic expansion. However, this rapid and unplanned growth, combined with population migration, likely created challenges for the efficient delivery of health services. The lack of infrastructure and the uneven development between urban and rural areas may have contributed to diverse economic and sanitary conditions in the region^(25)^.

Until the 1970s, Rondônia was an underdeveloped Federal Territory, with a sustainable economy based on extractive activities^(26)^. As economic growth advanced, health needs and the demand for nursing professionals increased. By the 1970s, the state already had hospital care services, emergency care, and health posts, such as those for childcare and tuberculosis. However, nurses working in the hospital sector represented only 4% of the workforce, while nursing assistants and attendants accounted for 57%^(27)^.

The Dr. Ludma Frota Childcare Post, founded on October 29, 1948, was a specialized facility for child care, offering Laboratory services, Prenatal Hygiene, Child Hygiene, a Milk Kitchen, a Canteen, and a Dental Office. This Post was deactivated in 1975 for renovations and was later transformed into the Federal Health Office^(28)^.

The Darcy Vargas Maternity Hospital, founded in 1948, represented a significant milestone in the expansion and improvement of maternal and child health services, since maternal and child mortality was a public health problem considered a federal priority.

The São José Hospital was founded in 1929 and became the largest and main health institution serving the population of Rondônia. Initially, it had about 60 beds and provided care to the sick from the Madeira River region and more remote areas along the Madeira Mamoré Railway (*EFMM*)^(27)^. The hospital had an Outpatient Clinic and a Dispensary (procedure room), with three operating rooms, a temporary stay room for patients with three beds, and a children’s room with one bed, in addition to its own pharmacy^(27)^. It operated in conjunction with the home care service of Porto Velho, where a nurse was assigned weekly to perform dressings and distribute medications in the Guajará Mirim region. In addition, there were Emergency Posts, the main ones located in Santo Antônio do Rio Madeira, Jacy Paraná, Presidente Marques or Abunã, as well as two dispensaries in different areas of the city of Porto Velho^(27)^.

In 1932, two more pavilions of the São José Hospital were inaugurated next to the first building, along with another pavilion for infectious diseases. Later, the Maternity unit and the tuberculosis ward were built^(26)^. That year, the Hospital’s Clinical Staff consisted of one physician and seven assistants, who annually attended approximately 4,138 adults and 520 children, offering services such as consultations, prescriptions, dressings, and injectable applications^(27)^.

The former Dr. Jayme Aben-Athar Sanatorium, currently known as the Santa Marcelina Community, was established to house individuals with leprosy, in accordance with the guidelines of Brazilian health policies in the first half of the twentieth century. At that time, people suspected of having leprosy - considered a ‘threat’ to the population due to the potential for disease transmission - were segregated in locations far from urban centers, which required nursing labor for their operation^(29)^. The inauguration took place on September 13^th^, 1954, on the anniversary of the creation of the Federal Territory of Guaporé, and it became known as the ‘Aben-Athar Colony,’ in honor of the public health physician who dedicated his life to the study of leprosy^(30)^. In 1975, administration of the community was transferred to the Sisters of Santa Marcelina. They established a philanthropic hospital that also offered private services at prices lower than those practiced in the market^(29)^.

The advertisement from Drugstore Avenida reflects the role of the nurse in administering medications - specifically injectables - in this type of establishment, highlighting the expression ‘experienced nurse,’ used to lend credibility to the service and attract clientele.

Another role of the nurse publicized in the newspaper was that of health educator, clearly illustrated in the article ‘Your Child,’ in which a mother observes the nurse wrapping the baby in swaddling cloths without leaving wrinkles or folds, thus preventing damage to the skin’s integrity. Health education has been a foundational component of public health nursing training in Brazil since the creation of the School of Nurses of the National Department of Public Health in Rio de Janeiro in 1923, when one of the requirements for admission to the program was that the candidate hold a Normal School diploma^(31)^. Training as a primary school teacher ensured that future nurses had the pedagogical skills necessary to educate the population about disease prevention and health promotion.

The data represent the social image of the nurse, which is influenced by stereotypes, signs, and symbols that reflect the socio historical construction of the profession, shaped by gender, religious, and technical factors.

Nationally, during the military regime in Brazil (1964 - 1985), Nursing was impacted by the 1968 University Reform, which increased the admission of men into the program through the unified entrance exam, as well as by government directives aimed at controlling and regulating the profession. Despite this, women continued to play a crucial role in the health sector, facing challenges and contributing significantly to improving population health, especially during health crises, while remaining the predominant workforce^(32)^. During this same period, the feminist movement influenced nurses to seek greater recognition and equal rights, leading to the emergence of labor unions and professional associations aimed at improving working conditions and salaries^(33)^.

The first mention of Nursing Technicians in Alto Madeira newspaper was identified only in 1976. Authorization for secondary level professional training in fields beyond agriculture, industry, and commerce occurred in 1962, following the enactment of the National Education Guidelines and Framework Law. The first Nursing Technician program was created in 1966 at the Anna Nery School of Nursing of the University of Brazil, with support from the *Associação Brasileira de Enfermagem* (*“ABEn”*, Brazilian Nursing Association)^(26)^.

Also at the national level, in the 1970s, *ABEn* - together with university nursing programs and professionals across the country - advanced the movement for the creation of a regulatory body for the profession, resulting in the establishment of the Federal and Regional Nursing Councils System through Law No. 5,905 of 1973. In 1972, the first Nurses’ Union had been created in Rio Grande do Sul, illustrating the dynamism of that decade in the field of professional development. However, nursing education was not uniform across all regions and was considered insufficient to meet the health needs of the Brazilian population. Therefore, due to the expansion and increasing complexity of health services in the 1970s - a decade marked by the construction of modern hospitals - news articles highlighting the importance of nursing frequently appeared in the press.

The Regional Nursing Council of Rondônia (*Coren RO*) was inaugurated in Porto Velho as a branch office in 1979, under the responsibility of Nurse Lucinda Shookenes Bentes^(34)^. Since then, the entity has carried out its role of professional registration and oversight in the state. Before that, nursing professionals working in Rondônia were linked to *Coren Amazonas* (AM).

The analytical perspective of these data allows us to infer the existence of diametrically opposed phenomena - on one hand, the precarization of labor, and on the other, the valorization of female workers as a political strategy to publicly attract women to fill workforce needs driven by market demand.

The greatest emphasis given to Nursing in Alto Madeira newspaper occurred in 1976, when the first Nursing Week in Rondônia took place. Organized by the state government and *Coren AM*, it included the participation of authorities and prominent professionals from medicine and nursing, as well as opening and closing ceremonies, a course offering, lectures, and social activities. The venues chosen to host Nursing Week were a Normal School and the Public Library. The choice of the Normal School may have attracted young women to the nursing profession, recalling one of the admission requirements of the country’s early modern nursing schools.

In the first Nursing Week of Rondônia, one can observe the efforts of the newly created *Coren* to elevate the status of nursing in the state. With articles published daily from May 11^th^ to the 21^st^ 1976, the newspaper informed the population about the nursing profession and revealed the political interest of government officials, business leaders, and physicians in increasing the number of workers, giving special visibility to the professional nurse - even if with some divergence of interests typical of a profession still in the process of consolidation. In the physician’s lecture, the importance of the nurse as a supplement to medical work was emphasized; in the nurse’s lecture, the focus was on professional regulation; and in the mayor’s speech, on humanitarian attributes. The identity of the nurse was still intertwined with that of assistants and religious workers, except in the voices of the nurses themselves.

The data point to the recognition, by agents of the Brazilian State, of the importance of the professionalization of nursing and of its capacity to positively influence the management and delivery of health systems and services.

Throughout the second half of the twentieth century in Brazil, the nursing profession was influenced by the empowerment of women driven by feminist movements, and, with the country’s process of redemocratization, nurses gradually gained greater autonomy, especially after the creation of the *Sistema Único de Saúde* (*“SUS”*, Unified Health System). Over the decades, nursing became a profession predominantly occupied by women, reflecting social expectations associated with care and assistance - roles traditionally attributed to women^(35)^.

Recognizing the phenomenon of the feminization of the profession, nurses faced challenges stemming from the stereotyped view of their occupation as an extension of responsibilities traditionally assigned to women. In the first half of the century, they often confronted unfavorable working conditions, including long shifts and low pay^(36)^. Motivated by the transformations brought about by the country’s economic and scientific development, they organized themselves into councils, unions, and associations to advocate for improved working conditions and the professional recognition they needed. These efforts led to advances such as the creation of graduate programs (stricto sensu) in 1972 and the enactment of the Professional Practice Law in 1986, both of which played a crucial role in consolidating and advancing this essential field within Brazilian health care^(37)^.

Due to the absence of nursing programs during the period studied, a slow and gradual process of professionalization of Nursing in Rondônia was observed. Nursing workers were present in the main institutions throughout the period, although in insufficient numbers until Nurses and Nursing Assistants arrived in the state to improve the quality of care. It is evident that the growth in the number of trained workers enabled movements toward the consolidation of the profession, a process reinforced by the creation of *Coren RO*, which became responsible for registering professionals and encouraging the struggle for professional recognition, aiming to overcome the health challenges characteristic of Amazonian border regions.

### Study limitations

The scarcity of historical documents and scientific articles on the subject, as well as the lack of information produced by nursing in Rondônia, constitute limitations of this study, along with the limited detail available about the professionals mentioned in the newspaper analyzed. Additionally, these gaps characterize the research as a potential opportunity for future investigations in this field.

### Contributions for the practice

The historical analysis reveals fundamental and often overlooked aspects of the nursing profession. Understanding these elements not only enriches knowledge about the profession but also provides crucial insights for the continuous improvement of Nursing, highlighting its vital role in health promotion and population care. It contributes to new studies focused on oral histories, comparisons with other regions, detailed analyses of legislative changes, and evaluations of the impact of specific health policies, all of which have the potential to enrich and deepen the understanding of the history of nursing in Rondônia.

## FINAL CONSIDERATIONS

Nursing in Rondônia, between 1946 and 1976, remained largely silenced within the political sphere of health until the 1970s, mainly due to the influence of the national political context. The transformation observable through the sources found in Alto Madeira newspaper includes the increasing mention of these workers and the praise of their qualities, including experience and competence.

Female nurses were cited more frequently in the newspaper, including by name, influenced by the educational model that began as exclusively female and lasted from 1931 to 1949, as well as by the need for public recruitment of these professionals to implement public health policies and meet the demands of the hospital context at the time. Consultant nurses, nursing students, and representatives of professional organizations gained visibility and space in the newspaper, culminating in the full coverage of the 1976 Nursing Week.

A transition from traditional practices to a more scientific and institutionalized model can be observed, driven by social and political changes that reached the region later than in other Brazilian states. The professional identity of nursing personnel strengthened through technical and scientific training in public health and hospital care, even before higher education programs existed in Rondônia.

Despite challenges such as precarious conditions and social stigma, nurses played an essential role in promoting the health of the population of Rondônia, a contribution rooted in the centrality of nursing’s.

## Data Availability

The research data are available within the article.
