# Supplementary material for: Nursing Practice in Rondônia (1946-1976): a historical study based on the Alto Madeira Newspaper
Source: Rev Bras Enferm. 2026 Oct 19;79:e20250309. doi: 10.1590/0034-7167-2025-0309 (PMC13679596; doi:10.1590/0034-7167-2025-0309)
Supplement: Translation

# Translation: Atuação da Enfermagem em Rondônia (1946-1976): um estudo histórico a partir do Jornal Alto Madeira

**DOI:** 10.1590/0034-7167-2025-0309pt

**Authors:** Phernando Pereira dos Santos, Jackeline Felix de Souza, Maria Lígia dos Reis Bellaguarda, Deybson Borba de Almeida, Maria Sagrario Gómez Cantarino, Maria Angélica de Almeida Peres

**Affiliations:** I Universidade Federal do Rio de Janeiro. Rio de Janeiro, Rio de Janeiro, Brasil; II Universidade Federal de Rondonópolis. Rondonópolis, Mato Grosso, Brasil; III Universidade Federal de Santa Catarina. Florianópolis, Santa Catarina, Brasil; IV Universidade Estadual de Feira de Santana. Feira de Santana, Bahia, Brasil; V Universidad de Castilla-La Mancha. Toledo, Espanha

**Keywords:** Enfermagem, Profissionais de Enfermagem, Jornal, Cuidados de Enfermagem, História da Enfermagem.

## Abstract

**Objetivos:**

analisar a atuação dos trabalhadores de Enfermagem em Rondônia entre 1946 e 1976.

**Métodos:**

estudo sócio-histórico, qualitativo e documental, baseado na análise de matérias do Jornal Alto Madeira. A pesquisa documental seguiu três etapas: pré-análise, organização e análise dos dados, com interpretação ancorada no contexto sócio-histórico da enfermagem no estado.

**Resultados:**

observou-se aumento progressivo no engajamento profissional da enfermagem, com destaque para contratações, locais de atuação, anúncios e eventos. A visibilidade feminina foi significativa, ressaltando-se qualidades como experiência e competência. A atuação abrangeu cuidados, apoio médico, trabalho em equipe e educação em saúde.

**Conclusões:**

apesar das condições precárias de trabalho e da ausência de cursos de enfermagem no período, a atuação desses profissionais foi registrada pelo Jornal Alto Madeira, que serviu como importante fonte histórica sobre a profissão e a consolidação da enfermagem em Rondônia, especialmente após a criação do Conselho Regional de Enfermagem na região Amazônica.

## INTRODUÇÃO

A produção de estudos sobre a História da Enfermagem na região Norte do Brasil vem crescendo lentamente, sendo este fato atribuído às características do próprio desenvolvimento da saúde local, bem como às características populacionais^(1)^. A cultura indígena, que por muito tempo foi prevalente na região Norte, manteve nas aldeias suas práticas de saúde, evitando acessar serviços nos centros urbanos, e a população urbana cresceu mais significativamente ao longo do século XX.

O território de Rondônia pertenceu aos estados do Amazonas e Mato Grosso e, em 1943, foi designado como Território Federal do Guaporé, evoluindo para o Território Federal de Rondônia, em 1956. Tornou-se um estado em 1981. Esse processo teve impactos significativos no crescimento populacional e nas atividades econômicas, resultando em desafios ambientais como o desmatamento e a pressão sobre os recursos naturais^(2)^. Do mesmo modo, exigiu organização dos serviços de saúde pública e a presença de pessoal de enfermagem.

O desenvolvimento da saúde em Rondônia foi influenciado por diversos fatores. Nas décadas de 1900 a 1950, as condições geográficas e climáticas desafiadoras da região Amazônica, aliadas ao acesso limitado a essa região, possivelmente impactaram a oferta de serviços de saúde^(3)^. As populações indígenas e ribeirinhas sofreram com desafios específicos quando se iniciou a exploração econômica pela extração de látex^(4)^.

Foi no início do século XX, durante o Ciclo da Borracha, que se deu a construção da Estrada de Ferro Madeira-Mamoré (EFMM), que induziu a criação do primeiro hospital do estado, Hospital da Candelária, em 1907. Inicialmente destinado a atender os trabalhadores da EFMM e estrangeiros responsáveis pelo projeto, seu funcionamento deu-se até 1930, quando a estrada de ferro foi desativada^(5)^. Com seu fechamento, o Hospital São José, inaugurado em 1929, tornou-se referência ao atendimento da população, sendo um marco no atendimento à saúde da região por mais de cinco décadas^(5)^.

Estudo anterior que se debruçou sobre a enfermagem rondoniense de 1907 até 1930 apresenta que o perfil dos trabalhadores de Enfermagem no estado de Rondônia era constituído por civis, muitos deles imigrantes vindos de países como Marrocos, Líbano, Síria, Japão, China, Índia, Barbados, Bolívia, Itália e Alemanha, além de brasileiros de diversas regiões do país, a maioria do Nordeste. Eles atuavam no combate às doenças infectocontagiosas que assolavam a região, conduzidos pelas orientações dos médicos^(5)^.

Dentre os acontecimentos atrelados ao desenvolvimento econômico de Rondônia após a década de 1940, pode-se citar a criação de estradas tais como a BR 364, o surgimento de novas unidades de atendimento à saúde, e a criação do Conselho Regional de Enfermagem (Coren), na década de 1970, estabelecendo requisitos legais, incluindo padrões éticos, de formação e competências para a Enfermagem^(6)^.

O conhecimento produzido, além de divulgar a história da enfermagem em um estado onde tal produção é rara, contribuirá para a formação em Enfermagem por se tornar conteúdo de ensino capaz de trazer visibilidade à enfermagem nortista, cujo trabalho contribui para a elevação do índice de desenvolvimento humano na região pela prestação de cuidados e promoção da saúde.

## OBJETIVOS

Analisar registros da presença e atuação de pessoal de enfermagem durante o desenvolvimento urbano do estado de Rondônia.

## MÉTODOS

### Aspectos éticos

Esta pesquisa seguiu as diretrizes do *Standards for Reporting Qualitative Research* (SRQR) e, por se tratar de pesquisa em fontes disponibilizados pela Biblioteca Nacional ao público em geral, a submissão e aprovação por Comitê de Ética em Pesquisa não se aplica.

### Tipo de estudo

Pesquisa descritiva, qualitativa, exploratória, embasada pela História-Social.

### Contexto e cenário

O recorte espacial compreende o antigo Território Federal do Guaporé, situado na região Norte do Brasil, criado em 1943 por meio do Decreto n° 5.812 do mesmo ano. Em 1956 tornou-se Território Federal de Rondônia, que foi elevado à condição de Estado de Rondônia pela Lei n° 41 de 1981.

O recorte temporal do estudo, 1946-1976, corresponde à primeira e última menção a trabalhadores de enfermagem no Jornal Alto Madeira enquanto ele circulou como periódico do Território Federal, primeiro do Guaporé (1943-1955) e depois de Rondônia (1956-1980). Optou-se por manter o nome atual (Rondônia) no texto para facilitar ao leitor a identificação da região estudada.

### Fontes de dados

O corpus documental foi constituído por matérias do Jornal Alto Madeira, criado em 1917 como publicação bissemanal, gerenciado pelo comerciante João Soares Braga, um português que também foi tesoureiro do município devido às suas habilidades para a contabilidade. Uma análise da sua primeira edição o classificou como um Jornal de temática livre^(7)^. As correspondências enviadas ao Jornal eram selecionadas pelo seu gerente com o aviso de que “a redação não se responsabilizaria por opiniões emitidas por seus colaboradores”^(7)^. Circulou até 2017, ano do seu centenário, quando emitiu sua última edição.

### Procedimentos de coleta de dados

O método da pesquisa documental foi a escolha para tratamento das fontes e dados, constituído de três fases: pré-análise (seleção e reunião de documentos), organização do material (distribuição dos documentos e extração de sentido e significado das mensagens das fontes) e análise dos resultados (em que há a contextualização sócio-histórica, relações contemporâneas e respeito ao tempo histórico espaço-temporal)^(8,9)^.

Foram realizadas buscas de todas as edições em documentos disponíveis no site da Hemeroteca Digital Brasileira, a partir dos descritores “enfermeira”, “enfermeiro”, “enfermagem”. Foram identificadas 50 edições do Jornal Alto Madeira (RO) publicadas no século XX, com 39 fontes que atenderam aos critérios da pesquisa. Como critérios de inclusão utilizaram-se matérias sobre trabalhadores de enfermagem de Rondônia e matérias de interesse regional e nacional sobre a profissão. Foram excluídas matérias com fatos sobre a enfermagem ocorridos em outros estados e países, matérias repetidas, citação aos trabalhadores de enfermagem fora do contexto de trabalho, oração e contos em que apareciam as palavras-chave.

Os dados foram organizados utilizando-se o programa *Microsoft Excel* ^®^ e incluíram informações como caracterização da fonte (data da coleta, data do registro da informação, autor do registro, tema da notícia, referência bibliográfica) e qualquer outra informação sobre a enfermagem no período. A partir da planilha foram preparadas figuras para facilitar a exposição dos achados.

### Procedimentos de análise de dados

A análise contemplou três dimensões inter-relacionadas: o meio de comunicação, cuja avaliação considerou aspectos como linha editorial, credibilidade e estratégias discursivas; a interpretação do leitor, na qual emergiram sentidos ancorados em repertórios socioculturais e posicionamentos individuais; o contexto social de produção da notícia, que elucidou as influências de fatores históricos, políticos e econômicos na construção e circulação da informação^(10)^.

Utilizaram-se fontes indiretas, como livros e artigos científicos, para a interpretação e discussão, levando-se em consideração o contexto de Rondônia e da Enfermagem no recorte temporal, estabelecendo-se interações de concordância, compreensão ou discordância entre os fatos^(11)^.

## RESULTADOS

Trinta e nove pessoas foram citadas nominalmente nas matérias do Jornal Alto Madeira (RO) entre 1946 e 1976, sendo trinta e cinco delas trabalhadores de Enfermagem e duas personalidades destacadas na história da profissão (Florence Nightingale e Anna Nery). Destes, 23 foram citados nas décadas de 1940 e 1950 (Figura 1) e 12 nas décadas de 1960 e 1970 (Figura 2).

**Figura 1:** Relação dos trabalhadores de Enfermagem mencionados no Jornal Alto Madeira nas décadas de 1940 e 1950, Porto Velho, Rondônia, Brasil, 2025

*Fonte: Jornal Alto Madeira. Rondônia (RO): Biblioteca Nacional Digital (BR); 1948 [citado 2025 Feb 16]. Disponível em: https://bndigital.bn.gov.br/hemeroteca-digital/.*

**Figura 2:** Relação dos trabalhadores de Enfermagem mencionados no Jornal Alto Madeira nas décadas de 1960 a 1970, Porto Velho, Rondônia, Brasil, 2025

*Fonte: Jornal Alto Madeira. Rondônia (RO): Biblioteca Nacional Digital (BR); 1960-1970 [citado 2025 Fev 16]. Disponível em: https://bndigital.bn.gov.br/hemeroteca-digital/.*

O ano de 1976 apresentou maior número de matérias citando a enfermagem nos textos Jornalísticos. Em relação ao gênero, foram citados como pessoal de Enfermagem 12 pessoas do gênero masculino (34,29%) e 23 do gênero feminino (65,71%). É importante este destaque para demonstrar a presença de homens atuando na enfermagem mesmo após a implantação da Enfermagem Moderna no país, até então, exclusividade feminina.

As categorias citadas nas matérias incluíram Enfermeiro(a), Auxiliar de Enfermagem, Técnico de Enfermagem, Enfermeiro Prático, Enfermeira Diplomada, Praticante de Enfermagem, Assistente de Saúde e Estudante de Enfermagem.

Quanto aos locais onde havia atuação da enfermagem, as fontes permitiram o levantamento de alguns serviços. A Divisão de Saúde do Território do Guaporé/Rondônia contratava enfermeiros que eram lotados nos serviços públicos, de modo que ela não foi citada na Figura 3, que se refere apenas aos serviços de saúde citados nas matérias incluídas na pesquisa.

**Figura 3:** Locais de trabalho dos trabalhadores de enfermagem no estado de Rondônia mencionados no Jornal Alto Madeira entre os anos de 1946 e 1976, Porto Velho, Rondônia, Brasil, 2025

*Fonte: Jornal Alto Madeira. Rondônia (RO): Biblioteca Nacional Digital (BR); 1946-1976 [citado 2025 Fev 16]. Disponível em: https://bndigital.bn.gov.br/hemeroteca-digital/.*

Os locais identificados como cenários de atuação dos trabalhadores de enfermagem foram as seguintes instituições existentes em Porto Velho: Hospital São José, Posto de Puericultura, Maternidade Darcy Vargas, Sanatório Colônia Dr. Jayme Aben-Athar e Drogaria Avenida.

Os contextos de citação nominal do pessoal de enfermagem foram variados, como funções exercidas, designação para cargos e serviços, aniversário, anúncio, elogios, boas-vindas, dentre outros. A primeira notícia, de 1946, se refere às boas-vindas para a Enfermeira Zuleika Reis, que retornara da Capital Federal, onde fora gozar de licença. Na notícia, ela foi adjetivada de “competente enfermeira do Hospital São José”^(12)^. No mesmo ano, uma propaganda de enfermeira foi publicada, na qual Sebastiana Azevedo, “diplomada pelo Departamento de Saúde do Território”, oferece seus serviços em domicílio, a qualquer dia, a qualquer hora, e gratuitamente, às pessoas pobres que não pudessem se locomover até os serviços de saúde^(13)^.

Em relação às matérias cuja referência à enfermagem não era nominal, seus conteúdos abordavam educação em saúde, quadro de funcionários, regulamentação da profissão, entre outros. Em destaque, conforme apresentado na Figura 4, uma matéria intitulada “Teu Filho”, onde a imagem da enfermeira de branco e usando touca é divulgada em atividade de educação em saúde.

**Figura 4:** Matéria “Teu Filho” publicada no Jornal Alto Madeira, Porto Velho, Rondônia, Brasil, 1948

*Fonte: Jornal Alto Madeira. Rondônia (RO): Biblioteca Nacional Digital (BR); 1948 [citado 2025 Fev 16]. Disponível em: https://bndigital.bn.gov.br/hemeroteca-digital/.*

Uma propaganda da Drogaria Avenida divulgava “aplicação de injeções a preços populares por enfermeiro competente”^(14)^.

Outra notícia que foi destaque no Jornal Alto Madeira, em 1976, relatava a vinda de estudantes de enfermagem da Universidade Federal do Rio Grande do Sul (UFRGS) para participar de uma operação nacional do Projeto Rondon que visava prover reforços na área da saúde. As estudantes atuaram em 23 localidades de Guajará Mirim/RO até Vila Bela/MT. A notícia trazia a seguinte redação:

“As jovens enfermeiras, além da administração de medicamentos e curativos, deram orientação em cada comunidade a pessoas capazes de assumir tal tipo de atividade, e orientaram as mães sobre problemas de puericultura”^(15)^.

A Semana da Enfermagem foi destaque no Jornal Alto Madeira em 12 de maio de 1976, com a matéria “Governador preside a abertura da Semana da Enfermagem”. A abertura realizada pelo governador Humberto da Silva Guedes foi no Colégio Normal Carmela Dutra, às 20h. A Semana foi promovida pela Secretaria de Saúde e Ação Social do Território e pelo Conselho de Enfermagem. A matéria anuncia que no dia 13 de maio seria proferida palestra sobre a Organização Administrativa da Secretaria de Saúde pelo Dr. Humberto Vieira Leal e que, no dia 15, a enfermeira Marlene Clomone faria a exposição do tema “A participação da Enfermagem no Plano de Saúde de Rondônia”^(16)^.

Durante toda a Semana da Enfermagem, matérias foram publicadas em sequência nas edições do Jornal Alto Madeira. No dia 13, a matéria tinha o mesmo título da anterior, “Governador preside a abertura da Semana da Enfermagem”, descrevendo a solenidade e dando detalhes da programação, que contava com palestras, composição da Diretoria do Conselho de Enfermagem (Coren), culto, missa, elaboração de estudos e o Programa de Integração Rural do Plano de Saúde de Rondônia^(17)^.

No dia 14, a matéria teve como título: “Governador destaca função da Enfermeira”. No dia 15, “Enfermeira também é importante na cura do doente” e, na edição de domingo e segunda-feira, 16 e 17 de maio, saiu a matéria “Enfermagem”, na qual são nominalmente citadas as autoridades presentes na abertura, bem como a síntese de seus discursos. Destaca-se a presença do Governador do Território, do Prefeito, do Secretário de Saúde e Ação Social, do Secretário de Educação, do Diretor do Departamento de Recursos Humanos da Superintendência do Desenvolvimento da Região Centro-Oeste (SUDECO) e do Diretor da Complano (Consultoria de Planejamento e Organização)^(18)^.

As falas citadas foram: a do Secretário de Saúde e Ação Social, Jacob Atallah, “que falou da importância da Enfermagem no contexto da atividade médica, como um suplemento indispensável a esta atividade”; da Consultora Carmen das Graças Lozich, “representante do Conselho Regional de Enfermagem, que esclareceu sobre a regulamentação da profissão de Enfermeira”, da Consultora de Enfermagem da Complano, Marlene Costa Câmara, “que destacou o trabalho da Enfermeira que dignifica a profissão, ressaltando a condição da mulher enfermeira, digno de ser reconhecido” e, por fim, a do Governador Humberto da Silva Guedes que “encerrou a solenidade pondo em relevo o trabalho da enfermeira como um serviço alicerçado no respeito à dignidade humana, onde o carinho e o conforto ao necessitado de assistência é fundamental”^(19)^.

No seguimento da Semana da Enfermagem, nos dias 18, 19 e 20 de maio de 1976, matérias convidavam e anunciavam o encerramento do evento, conforme ilustrado da Figura 5.

**Figura 5:** Matérias sobre o encerramento da Semana da Enfermagem em Rondônia, publicadas nas edições de 18, 19 e 20 de maio de 1976 no Jornal Alto Madeira, Porto Velho, Rondônia, Brasil, 1976

*Fonte: Jornal Alto Madeira. Rondônia (RO): Biblioteca Nacional Digital (BR); 1976 [citado 2025 Fev 16]. Disponível em: https://bndigital.bn.gov.br/hemeroteca-digital/.*

Nos dias em que o convite para o encerramento da Semana da Enfermagem foi publicado, também saíram matérias sobre a enfermagem. Destaca-se a informação sobre o projeto do deputado Nelson Carneiro que acrescentaria dispositivos à Lei n° 2.604/1955 que regulava o exercício da enfermagem profissional no Brasil, no qual “os enfermeiros práticos em exercício por mais de cinco anos, depois de teste de conhecimento receberão - se aprovados - o diploma oficial de enfermagem”. Outra matéria informa que ocorreu a oferta do Curso de Administração em Saúde dentro da Semana da Enfermagem, e faz convite à classe médica, às autoridades e ao povo para comparecimento à sessão de encerramento^(20)^.

Na sequência, a matéria do dia 21 de maio tem o título “Governador presidiu ontem o Encerramento da Semana da Enfermagem” e, por fim, no dia 22 de maio, saiu na coluna “Destacando” que o Dr. Leônidas Rachid Jaudy recebeu enfermeiros e convidados em seu sítio, comemorando a Semana da Enfermagem, contando com a presença do Secretário de Saúde, Dr. Jacob Freitas Atallah.

## DISCUSSÃO

Ao longo do século XX, no Brasil, a profissão de Enfermagem passou por uma notável evolução em relação à organização profissional, quando foram estabelecidos cursos de formação em enfermagem, como o Curso para a Formação de Enfermeiras, em São Paulo, em 1912, o Curso de Enfermeiras Voluntárias, no Rio de Janeiro, em 1914, ambos da Cruz Vermelha Brasileira (CVB), e o Curso de Enfermeiras do Departamento Nacional de Saúde Pública, em 1923, na capital, Rio de Janeiro. Na década de 1930 teve início o curso de auxiliar de enfermagem, e na de 1960 o de técnico de enfermagem. As enfermeiras diplomadas (nível superior) criaram uma Associação e foram paulatinamente assumindo funções de liderança, gestão e tomada de decisões nas instituições de saúde pública e hospitalares, ao tempo em que ocorria o distanciamento dos religiosos não diplomados das ações de cuidado, movimento que foi mais intenso na região Sudeste do país, e que culmina com a regulamentação da profissão nas décadas de 1970-80^(21,22)^.

É importante destacar que o Jornal Alto Madeira publicava matérias onde pessoas eram identificadas como enfermeiros de acordo com a contratação, e não com o nível de formação. No período de 1931 a 1949 esteve em vigor o decreto lei n° 20.109/1931 que regulava o exercício profissional da enfermagem no Brasil. O artigo 1° deste decreto apresentava a seguinte redação:

*Art. 1º Só poderão usar o título de enfermeiro diplomado ou enfermeira diplomada ou as iniciais correspondentes a estas palavras: a) os profissionais diplomados por escolas de enfermagem oficiais ou equiparadas na forma da presente lei; b) os profissionais que, sendo diplomados por escolas estrangeiras reconhecidas pelas leis do seu país, se habilitarem perante a banca examinadora competente ou forem contratados pela administração federal ou estadual* ^(23)^.

De fato, isto ocorreu posteriormente na região Norte, pois sem a presença de curso de formação em enfermagem até 1944, quando foi criada a Escola de Enfermagem do Pará, foi mantida a denominação enfermeiro para qualquer praticante de enfermagem ou auxiliar hospitalar, com curso preparatório ou não, especialmente em locais onde não havia escola de enfermagem.

Quanto ao gênero, há uma predominância feminina quando o Jornal se refere nominalmente à enfermeiros(as). Duas trabalhadoras foram citadas em 1946, aumentando para dez em 1976. A presença masculina surge de forma incipiente com um trabalhador em 1955 e um em 1976, refletindo uma disparidade de gênero significativa. Em 1955 apareceu citação a cinco trabalhadores homens. Esse cenário destaca a relação histórica da enfermagem ao papel social feminino. O aumento de mulheres na profissão reflete tanto a crescente demanda por profissionais de saúde quanto a maior inserção feminina no mercado de trabalho, impulsionada por mudanças sociais e econômicas do período, e mais ainda pela implantação da Enfermagem Moderna no país, cuja característica inicial era a exclusividade feminina.

Em 1946, um anúncio oferecendo serviços de enfermeira diplomada em Rondônia foi divulgado. Contudo, este termo era usado como identidade da enfermeira formada por escola dentro de um padrão de ensino instituído no país desde 1931, o que carece de esclarecimento, uma vez que a historiografia da enfermagem não registra escola padrão na região Norte no período. Contudo, sabe-se que o Jornal estava alinhado ao pensamento hegemônico da época, pelo qual o diploma não era a principal exigência para se tornar trabalhador de enfermagem em locais onde se tinha carência desse pessoal.

Em relação aos profissionais técnicos e auxiliares, os resultados apontaram maior referência à auxiliares de enfermagem no ano de 1950. Apesar de a regulamentação do curso de auxiliar de enfermagem no Brasil ter ocorrido em 1949, o cargo de auxiliar já existia nos serviços de saúde^(24)^.

No final da década de 1960, observou-se a implementação de medidas de colonização, levando à ocupação gradual da região Norte, quando Rondônia vivenciou uma expansão econômica expressiva. Contudo, esse rápido crescimento desordenado, aliado à migração populacional, provavelmente trouxe desafios para a entrega eficiente de serviços de saúde. A falta de infraestrutura e o desenvolvimento desigual entre áreas urbanas e rurais podem ter contribuído para condições econômicas e sanitárias diversas na região^(25)^.

Até os anos de 1970 Rondônia era um Território Federal pouco desenvolvido, com uma economia sustentável baseada em atividades extrativistas^(26)^. À medida que o crescimento econômico avançava, aumentavam as necessidades de saúde e a demanda por profissionais de Enfermagem. Na década de 1970, o estado já possuía serviços de atenção hospitalar, pronto socorro e postos de saúde, como os de puericultura e tuberculose. Entretanto, os enfermeiros que atuavam no setor hospitalar representavam apenas 4% da força de trabalho, enquanto os auxiliares de enfermagem e atendentes compunham 57%^(27)^.

O Posto de Puericultura Drª Ludma Frota, fundado em 29 de outubro de 1948, era um ambiente especializado para o atendimento infantil, oferecendo Laboratório, Higiene Pré-Natal, Higiene Infantil, Lactário, Cantina e Gabinete Dentário. Esse Posto foi desativado em 1975 para sofrer reformas e depois foi transformado na Delegacia Federal de Saúde^(28)^.

A Maternidade Darcy Vargas, fundada em 1948, representou um marco significativo na expansão e melhoria dos serviços de saúde materno-infantil, uma vez que a mortalidade materno-infantil era um problema de saúde pública que o governo federal considerava prioridade.

O Hospital São José foi fundado em 1929 e tornou-se a maior e principal instituição de saúde para a população rondoniense. Inicialmente, dispunha de cerca de 60 leitos e prestava atendimento aos enfermos da região do Rio Madeira e regiões mais afastadas da EFMM^(27)^. O Hospital contava com Ambulatório e Dispensário (sala de procedimentos), com três salas de operações, uma sala de estadia provisória de doentes com três leitos, e uma para crianças, com um leito, além de uma farmácia própria^(27)^. Operava em conjunto com o serviço de assistência domiciliar de Porto Velho, onde um enfermeiro era designado semanalmente para realizar curativos e distribuir medicamentos na região de Guajará-Mirim. Além disso, havia Postos de Pronto Socorro, sendo os principais em Santo Antônio do Rio Madeira, Jacy-Paraná, Presidente Marques ou Abunã, além de dois dispensários em locais distintos na cidade de Porto Velho^(27)^.

Em 1932 foram inaugurados mais dois pavilhões do Hospital São José, ao lado do primeiro edifício, e mais um pavilhão de moléstias infecciosas. Posteriormente, foram construídos a Maternidade e o setor de tuberculosos^(26)^. Neste ano, o Corpo Clínico do Hospital era composto por um médico e sete auxiliares que atendiam anualmente cerca de 4.138 adultos e 520 crianças, oferecendo serviços como consultas, receituários, curativos e aplicações de injetáveis^(27)^.

O antigo Sanatório Dr. Jayme Aben-Athar, atualmente denominado Comunidade Santa Marcelina, foi estabelecido para acolher portadores de hanseníase, em consonância com as diretrizes das políticas de saúde brasileiras da primeira metade do século XX. Naquela época, pessoas com suspeita de hanseníase, consideradas uma “ameaça” à população pelo potencial de transmitir a doença, eram segregadas em locais afastados dos centros urbanos, que precisavam de mão de obra de enfermagem para seu funcionamento^(29)^. A inauguração ocorreu em 13 de setembro de 1954, no aniversário da criação do Território Federal do Guaporé, e ficou conhecido como “Colônia Aben-Athar”, em homenagem ao médico sanitarista que dedicou sua vida ao estudo da hanseníase^(30)^. Em 1975, a administração da comunidade foi transferida para as Irmãs Marcelinas. Elas estabeleceram um hospital filantrópico, que também oferecia serviços particulares a preços inferiores aos praticados no mercado^(29)^.

O anúncio da Drogaria Avenida reflete o papel do enfermeiro na administração de medicamentos, no caso, injetáveis, nesse tipo de estabelecimento, com destaque para a expressão “enfermeiro experiente”, usada para dar credibilidade ao serviço prestado e atrair a clientela.

Outro papel da enfermeira divulgado no Jornal foi o de educadora em saúde, objetivamente ilustrado na matéria “Teu Filho”, onde a mãe observa a enfermeira colocando os cueiros na bebê sem deixar rugas ou dobras, prevenindo danos à integridade da pele. A educação em saúde sustentou a formação de enfermeiras de saúde pública no Brasil desde a criação da Escola de Enfermeiras do Departamento Nacional de Saúde Pública, no Rio de Janeiro, em 1923, quando uma das exigências para ingresso no curso era que a candidata tivesse diploma de Curso Normal^(31)^. A formação de professora primária garantia capacitação pedagógica da futura enfermeira para educar a população sobre prevenção de doenças e promoção da saúde.

Os dados representam a imagem social da enfermeira que é influenciada pelos estereótipos, signos e símbolos que remetem à construção sócio-histórica da profissão, atravessada pelas questões de gênero, religiosas e técnicas.

Nacionalmente, no período do regime militar no Brasil (1964-1985), a Enfermagem foi impactada pela Reforma Universitária de 1968, que aumenta a entrada de homens no curso pelo vestibular unificado, e pelas diretrizes governamentais que buscavam controlar e regulamentar a profissão. Apesar disso, as mulheres continuaram a desempenhar um papel crucial no setor de saúde, enfrentando desafios e contribuindo de maneira significativa para a melhoria da saúde da população, especialmente durante crises sanitárias, se mantendo como força de trabalho feminino^(32)^. Ainda nesse período, o movimento feminista influenciou as Enfermeiras a buscarem maior reconhecimento e igualdade de direitos, levando ao surgimento de movimentos sindicais e associações profissionais que visavam melhorar condições de trabalho e salários^(33)^.

A primeira menção a Técnicos de Enfermagem no Jornal Alto Madeira foi identificada apenas em 1976. A permissão para a profissionalização no segundo grau em áreas além da agrícola, industrial e comercial ocorreu em 1962, após a promulgação da Lei de Diretrizes e Bases da Educação Nacional. O primeiro curso de Técnico de Enfermagem foi criado em 1966 na Escola de Enfermagem Anna Nery, da Universidade do Brasil, com o auxílio da Associação Brasileira de Enfermagem (ABEn)^(26)^.

Ainda no cenário nacional, na década de 1970, a ABEn, juntamente com os Cursos de Enfermagem Universitários e profissionais de todo o país, ganhava a luta para a criação de um órgão regulamentador da profissão, com a implantação do Sistema Conselho Federal/Conselhos Regionais de Enfermagem pela Lei n°5.905 de 1973. Em 1972, o primeiro Sindicato de Enfermeiros havia sido criado no Rio Grande do Sul, o que mostra a efervescência desta década no campo do desenvolvimento profissional. Porém, a formação em enfermagem não era igualitária em todas as regiões, chegando a ser considerada insuficiente para atender a todas as demandas de saúde da população brasileira. Assim, devido ao aumento e à complexidade dos serviços de saúde na década de 1970, que foi marcada pela construção de hospitais modernos, notícias sobre a importância da enfermagem eram manchetes Jornalísticas.

O Coren-RO foi inaugurado em Porto Velho sob a configuração de delegacia, em 1979, tendo como responsável a Enfermeira Lucinda Shookenes Bentes^(34)^. Desde então, esta entidade passou a exercer sua finalidade de registro e fiscalização profissional nesse estado. Antes disso, os profissionais de enfermagem que atuavam em Rondônia eram vinculados ao Coren-Amazonas (AM).

A linha analítica destes dados nos permite inferir a existência de fenômenos diametralmente opostos que se apoiam - por um lado a precarização do trabalho, e ao mesmo tempo a valorização das trabalhadoras como estratégia política de chamamento público de mulheres para compor os quadros em prol da necessidade mercadológica.

O maior destaque dado para a Enfermagem no Jornal Alto Madeira foi em 1976 quando ocorreu a primeira Semana da Enfermagem em Rondônia. Organizada pelo governo estadual e pelo Coren-AM, teve participação de autoridades e de profissionais de destaque da medicina e da enfermagem, além de cerimônias de abertura e de encerramento, oferta de curso, palestras e atividades sociais. Os locais escolhidos para sediar a Semana da Enfermagem foram uma Escola Normal e a Biblioteca Pública. A Escola Normal como sede pode ter atraído moças para a profissão de Enfermeira, o que remete a um dos requisitos das primeiras escolas modernas do país.

Na primeira Semana da Enfermagem de Rondônia percebem-se os esforços do recém-criado Coren para valorizar a enfermagem no estado. Com matérias publicadas diariamente entre 11 e 21 de maio de 1976, o Jornal esclarece a população sobre a profissão de enfermagem, e mostra o interesse político de governantes, empresários e médicos em aumentar o número de trabalhadores, dando visibilidade especial à profissional enfermeira, ainda que com certa divergência de interesses comuns ao se tratar de uma profissão em processo de consolidação. Na palestra do médico é destacada a importância da enfermeira como complemento desse profissional; na da enfermeira, a regulamentação profissional, e na do prefeito seus atributos humanitários. A identidade da enfermeira ainda se mesclava com a de auxiliares e religiosas, exceto na voz das próprias enfermeiras.

Os dados remetem ao reconhecimento dos agentes do Estado Brasileiro face à importância da profissionalização da profissão e da sua capacidade profissional de implicar positivamente na gestão e execução de sistemas e serviços de saúde.

Ao longo da segunda metade do século XX, no Brasil, a profissão de Enfermagem foi influenciada pelo empoderamento das mulheres a partir de movimentos feministas, e do processo de redemocratização do país veio a conquista crescente de autonomia, especialmente após a criação do Sistema Único de Saúde. Com o passar das décadas, a enfermagem transformou-se em uma carreira majoritariamente ocupada por mulheres, refletindo as expectativas sociais vinculadas ao cuidado e à assistência, tradicionalmente atribuídos aos papeis femininos^(35)^.

Reconhecendo o fenômeno de feminilização da profissão, as enfermeiras enfrentaram desafios decorrentes da visão estereotipada de sua ocupação como uma extensão das responsabilidades tradicionalmente associadas às mulheres, enfrentando, na primeira metade do século, condições laborais frequentemente desfavoráveis, incluindo jornadas extensas e remuneração reduzida^(36)^. Impulsionadas pelas transformações ocasionadas pelo desenvolvimento econômico e científico do país, elas se uniram em conselhos, sindicatos e associações para pleitear melhorias nas condições de trabalho e o reconhecimento profissional necessário, obtendo avanços como a criação da pós-graduação *stricto sensu* em 1972 e a sanção da Lei do Exercício Profissional, em 1986, que desempenharam papel crucial na consolidação e progresso dessa área relevante para a saúde no Brasil^(37)^.

Devido à falta de cursos de enfermagem no período estudado observou-se um processo lento e gradual de profissionalização da Enfermagem em Rondônia. Os trabalhadores de enfermagem se fizeram presentes nas principais instituições durante o período estudado, em quantitativo insuficiente até que Enfermeiros e Auxiliares de Enfermagem chegassem ao Estado para qualificar o cuidado. Percebe-se que o crescimento do número de trabalhadores formados possibilitou movimentos para a consolidação da profissão, o que foi reforçado pela criação do Coren-RO, responsável pelo registro de profissionais e incentivo à luta pelo espaço profissional, visando a superação dos desafios de saúde característicos de regiões de fronteira amazônica.

### Limitações do estudo

A escassez de documentos históricos e de artigos científicos sobre o tema, e a carência de informações provenientes da Enfermagem rondoniense mostram-se limitações para este estudo e, também, o reduzido detalhamento sobre os profissionais citados no Jornal pesquisado. Adicionalmente, essas fragilidades caracterizam a pesquisa como potencial oportunidade para futuras investigações nesse campo.

### Contribuições para a prática

A análise histórica revela partes fundamentais e frequentemente negligenciadas da profissão de Enfermagem. A compreensão desses aspectos não apenas enriquece o conhecimento sobre a profissão, mas também oferece informações cruciais para o aprimoramento constante da Enfermagem, destacando seu papel vital na promoção da saúde e no cuidado à população. Contribui para novos estudos focados em relatos orais, comparações com outras regiões, análises detalhadas das mudanças legislativas e avaliações do impacto de políticas de saúde específicas que têm o potencial de enriquecer e aprofundar a compreensão da história da enfermagem em Rondônia.

## CONSIDERAÇÕES FINAIS

A Enfermagem em Rondônia, entre os anos 1946-1976, manteve-se silenciada no campo político da saúde até a década de 1970, principalmente por atravessamentos do contexto político nacional. A transformação possível de ser observada por meio das fontes encontradas no Jornal Alto Madeira incluem a crescente menção a esses trabalhadores e enaltecimento às suas qualidades, incluindo experiência e competência.

A mulher enfermeira teve mais citação no Jornal, inclusive nominal, influenciado pelo padrão de ensino que iniciou exclusivamente feminino e durou de 1931 até 1949, inclusive por questões da necessidade do chamamento público dessas profissionais, a fim de implementar políticas de saúde pública, e pela demanda do contexto hospitalar à época. Enfermeiras consultoras, estudantes de enfermagem, representantes de entidades de classe ganharam espaço e matérias no Jornal, culminando com a cobertura integral da Semana da Enfermagem de 1976.

Observa-se a transição de práticas tradicionais para um modelo mais científico e institucionalizado, impulsionada por mudanças sociais e políticas refletidas na região com atraso quando comparada com outros Estados brasileiros. A identidade profissional do pessoal de Enfermagem se fortaleceu a partir da formação técnica e científica nos campos da saúde pública e hospitalar, antes de existir cursos de nível superior em Rondônia.

Apesar dos desafios, como condições precárias e estigma social, os enfermeiros desempenharam um papel essencial na promoção da saúde da população rondoniense, e isto se deve à centralidade e objeto de trabalho da profissão que é o cuidado em saúde.

## Data Availability

Os dados de pesquisa estão disponíveis no corpo do artigo.
